# Launching continuous kangaroo mother care through participatory action research in Iran

**DOI:** 10.1186/s12913-023-09372-0

**Published:** 2023-05-04

**Authors:** Marzieh Mohammadi, Anne-Marie Bergh, Niloufar Sattarzadeh Jahdi, Leila Valizadeh, Mohammadbagher Hosseini, Sevil Hakimi

**Affiliations:** 1 Student’s Research Committee, Tabriz University of Medical Science, Tabriz, Iran; 2 Research Centre for Maternal, Fetal, Newborn and Child Health Care Strategies, University of Pretoria, Pretoria, South Africa; 3 Department of Midwifery, Faculty of Nursing and Midwifery, Tabriz University of Medical Science, Tabriz, Iran; 4 Department of Pediatric Nursing, Faculty of Nursing and Midwifery, Shahid Beheshti University of Medical Science, Tehran, Iran; 5 Pediatric Health Research Center, Tabriz University of Medical Science, Tabriz, Iran; 6 Department of Midwifery, Faculty of Nursing and Midwifery. Research Center of Psychiatry and Behavioral Sciences, Tabriz University of Medical Science, Tabriz, Iran

**Keywords:** Continuous kangaroo mother care, Premature infants, Implementation, Barriers to implementation, Participatory action research

## Abstract

**Background:**

This study describes the launching of a unit for continuous kangaroo mother care (KMC) in a teaching hospital (Taleghani) in Iran.

**Methods:**

We used a participatory three-stage action research approach to establish a unit for continuous KMC: design (needs identification and planning for change); implementation (and reflection); and evaluation (and institutionalization). As part of the design and implementation stages, individual and focus group interviews were conducted with mothers, physicians, nurses, other healthcare personnel and policy makers. The evaluation was done by means of a standardized tool specifically developed for monitoring progress with the implementation of KMC.

**Results:**

Four themes relating to potential barriers to implementation emerged from the analysis of the staff interviews, namely barriers associated with the mother, the father, the physician and the health system. Mothers’ experiences of barriers were grouped into five themes: personal discomfort, fear, healthcare provider attitudes and actions, infrastructure constraints and family matters. An implementation progress score of 27.05 out of 30 was achieved, indicating that the continuous KMC unit was on the path to institutionalization. Some of the gaps identified related to policies on resource allocation, the discharge and follow-up system, and the transportation of infants in the KMC position.

**Conclusion:**

The study findings indicated that participatory action research is a suitable method for studying the establishment of a continuous KMC unit. When action research is practiced, there is a prospect of turning knowledge into action in the real world.

## Background

Kangaroo mother care (KMC) is a healthcare strategy for premature and low birthweight infants that includes skin-to-skin contact between caregiver and infant, exclusive breastfeeding whenever possible, and appropriate follow-up after hospital discharge [1]. It is a highly effective, low-cost intervention that forms part of the care for preterm and low birthweight newborns across the globe [2]. In low- and middle-income countries, effective, affordable care is necessary if neonatal morbidity and mortality are to be reduced, especially among premature infants [3, 4]. Some of the basic benefits of skin-to-skin contact for the small neonate are thermal regulation and stabilization of physiological functions. KMC also promotes mother–infant attachment [5]. KMC is practiced intermittently (a number of hours per day) or continuously (ideally 24 h per day) [1]. With this method of care, the neonate is placed naked in an upright position on the mother’s bare chest between her breasts. The baby’s head is turned to the side in a slightly upright position (the ‘sniffing’ position) and the mother sits at a 30–60° angle, with the baby’s back covered by a blanket [6].

Although considerable progress has been made in the past 20 years with the reduction of infant mortality in Iran [7], many more changes will have to be made to the approach to the treatment and care of premature infants in order to ensure that the long-term effects envisaged by the Sustainable Development Goals are achieved [8]. There have been numerous recommendations on the inclusion of continuous KMC (C-KMC) services in all hospitals, which entails that mother and baby should not be separated, with the baby in the KMC position for at least 20 h per day. By 2018 no hospital in Iran had a C-KMC unit. The Bogota Declaration acknowledges KMC as the essential right of every newborn and states that KMC must be an integral part of managing the care of premature and term newborns [9]. There are many reasons why KMC is not applied as part of standard care in Iran and elsewhere, including barriers related to the health system (e.g. organizational and service-delivery factors), to health care providers (e.g. absence of guidelines and lack of training) and to parents (e.g. barriers in respect of resources, acceptance and cultural beliefs) [10–12]. The XIIth International Conference on KMC highlighted the following strategies for KMC implementation at a national level: specification of a minimum set of indicators for assessing country-wide KMC scale-up; integration of KMC into the health system and implementation in hospitals, with special attention to KMC transportation and the establishment of KMC follow-up systems for KMC babies; and KMC for the term newborn [2].

This study reports on the results of tracking implementation during the creation of a separate unit for providing C-KMC in a teaching hospital in Iran.

## Methods

The study was conducted through participatory action research based on the approach proposed by McNiff [13]. Action research combines research with action and in the healthcare and hospital environment it could help to improve the services offered and empower healthcare providers and healthcare users [14]. This approach enables more conscious decision making for practice change because it provides a better understanding of the problem and the reasons underlying the problem [15]. The researcher does more than report on findings; he or she engages with the participating individuals [16]. The most important attribute of participatory action research is the dynamic, cyclical nature of the process. The primary stages of all action research are design, implementation, evaluation and, finally, institutionalization of the action [13]. This paper reports on the first phase described in the protocol, namely the tracking of the implementation of C-KMC. The study protocol has been published [17].

### Setting

This implementation study was conducted with the participation of faculty members and personnel working at Taleghani Hospital in Tabriz, the capital of Azerbaijan-e-Sharghi Province in the northwestern part of Iran. The hospital is a university-affiliated center with a level III neonatal intensive care unit (NICU). The hospital has a high-risk pregnancy ward and deals with about 6,000 births annually.

### Study process

The study followed a three-stage repetitive cycle over 17 months – from April 2019 to December 2020. The study had to be interrupted for two months from January 2020 to March 2020 when the newly-established C-KMC unit was closed per intra-hospital instruction as a result of the coronavirus pandemic.

**Fig. 1:**
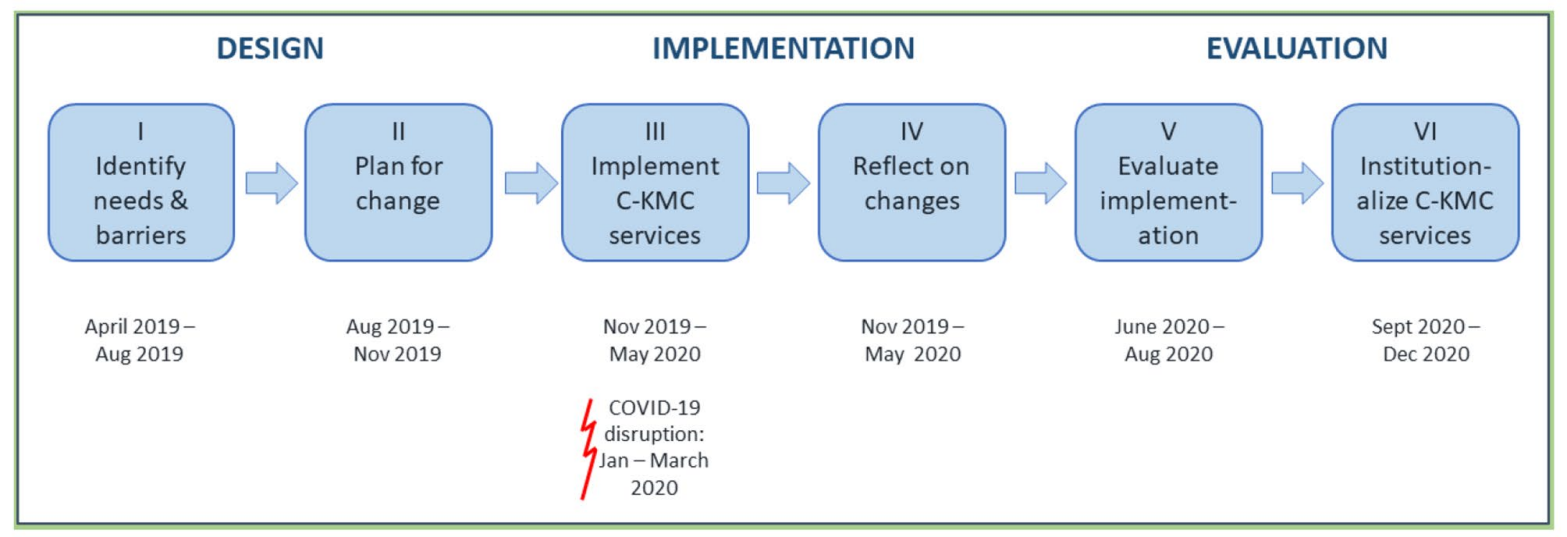
The action research process

The general stages of C-KMC implementation matched McNiff’s framework for action research, namely design; implementation; and evaluation. Every stage comprised two steps; in other words, the three general stages were divided into smaller components and implemented in six steps. More details pertaining to sampling, data collection and analysis are available in the study protocol [17]. Figure 1 gives a graphic depiction of the action research process.

The first stage (design) comprised two steps, namely identification of needs and planning for change. After obtaining formal permission for the implementation of the C-KMC program from the hospital director, the researchers collected information on the existing barriers to change by conducting semi-structured individual and focus group interviews with service providers, decision makers and mothers.

The second stage (implementation) consisted of two steps: implementation and reflection. Based on the findings from the first stage, the research team set out to eliminate the barriers and provide the required facilities and equipment. After the initial implementation, open reflection sessions were held with neonatal personnel every two weeks to identify weak points, problems and contingency issues with a view to deciding on the final modification of the program.

The third stage (evaluation) included the two steps of evaluation and institutionalization. The standardized KMC progress-monitoring tool (West African version 0.3, 2017) designed by Bergh and colleagues was used to evaluate the implementation of C-KMC [18]. This tool, which could serve as a checklist for identifying service gaps for further improvement, collects quantitative and qualitative data on the implementation of KMC. The tool has 18 sections; some are completed from self-reports by healthcare providers in the neonatal and KMC units and other individuals involved in the implementation process. The remaining items are completed from observations of the KMC and neonatal wards and the process of practicing C-KMC. The monitoring tool contains items which evaluate physical space, KMC practice, doctors`, nurses` and hospital managers` support for KMC, in-service orientation and training, et cetera. The maximum score on this checklist is 30. A score of 10 or more shows that the hospital is on the path to implementing KMC. A score of more than 17 indicates that the hospital is on the path to routinely offering KMC services and a score above 24 shows evidence that the care has been institutionalized [19]. Evaluation was performed by two authors from the research team (MM and SH). Some of the items depended on observation, so each evaluator rated these items after precise observations. Responses by the evaluators were compared afterwards. The items pertaining to documents were independently completed by two of the evaluators. This means that the validity of each document was assessed by both evaluators simultaneously.

### Data analysis

Data from focus group sessions and interviews were analyzed by means of inductive content analysis following a conventional approach; coding was derived directly from the text data [20]. Other information gathered by observing activities and reviewing existing documents was utilized in the reflection process and in making final modifications to the program.

Data collected with the aid of the progress-monitoring tool were captured on an Excel spreadsheet. An implementation progress score was calculated for the quantitative items contributing to the score. The rest of the information gathered was reported on in narrative form and used in further discussions of gaps and improvements needed.

## Results

### Step 1: identifying needs and barriers

The first step of this action research was a qualitative investigation aimed at identifying the barriers to the implementation of C-KMC with the participation of the healthcare providers and mothers. Three authors served as interviewers (MM, SH and NSJ). Five focus group discussions (FGDs) and four individual interviews were held with health care providers. These participants included 17 nurses, four midwives, efive neonatologists, the head of the hospital (pediatrician), the hospital manager (general physician), a matron (nurse) and the director of administrative affairs (with a postgraduate degree in hospital management). The results of provider perspectives have been reported on elsewhere [21]. We also conducted individual interviews with 13 mothers. The barriers to performing C-KMC are summarized in Table 1.

**Table 1:** Perspectives of service providers and mothers on the barriers to performing continuous KMC

Participant group	Theme	Category
Service-provider perspectives [21]	Barriers in relation to mother	‑ Cultural barriers ‑ Physical problems ‑ Psychological problems ‑ Personal hygiene ‑ Lack of orientation to KMC
	Barriers in relation to father	‑ Cultural issues ‑ Lack of support for mother ‑ Lack of support for father
	Barriers associated with physicians	‑ Cognitive barriers (reluctance) ‑ Material barriers (loss of income)
	Barriers associated with the system	‑ Financial barriers (user fees) ‑ Lack of facilities
Mothers’ perspectives	Personal discomfort	‑ Impatience ‑ Duration of KMC ‑ Alone in ward ‑ Physical weakness
	Fear	‑ Fear of falling asleep and harming the infant during KMC
	Healthcare provider attitudes and actions	‑ Lack of interest and collaboration by nurses ‑ Physicians’ visiting schedules
	Infrastructure	‑ Absence of armchair ‑ Absence of privacy
	Family matters	‑ Father does not support mother ‑ Lack of family support ‑ Other children at home

In general, the results indicated that emphasis should be placed on orientating parents to KMC, training physicians, nurses and other healthcare personnel, and providing facilities and equipment to enhance C-KMC practice. In the short term, there was a need to adjust patient fees and medical insurance in some cases. For example, one neonatologist complained that the tariff for accommodation in the C-KMC unit had not been clearly set. If the same rate were charged as for a normal infant bed this would not be acceptable to the physician leaders [21].

### Step 2: planning

Based on the findings of Step 1 regarding barriers, problems, priorities and solutions for the development of C-KMC, a short-term C-KMC operational program was drafted with a view to reaching agreement between the research team and the nursing participants. Two KMC courses with practical and theoretical components were offered for personnel engaged in neonatal care. A space for C-KMC, about 60 square meters in extent, was allocated inside the neonatal ward adjacent to the NICU and the necessary equipment and amenities were procured. Furthermore, following collaboration with the hospital’s financial unit, it was decided that the stay in the C-KMC unit would be less costly than the stay in the NICU.

### Step 3: implementing C-KMC services

The third step consisted of implementing the program. The C-KMC unit contained four comfortable beds, a closet, a dining table with four chairs, bookshelves with educational books and other amenities for the mothers’ comfort, such as a refrigerator, microwave, oven, kettle and television set.

Mothers with neonates eligible for KMC were educated in the practice of continuous KMC, which they applied in the unit.

### Step 4: reflecting on changes

This stage entailed two-weekly discussion sessions with personnel for three months to give feedback on progress with implementation and to identify problems and other contingent matters. The perspectives and ideas of the participants, especially mothers, were used to re-evaluate the program’s strengths and weaknesses; staff members proposed possible solutions to challenges. The reflections of participants and the solutions that were implemented are presented in Table 2.

**Table 2:** Problems in the C-KMC unit and implemented solutions

Problems brought up by nurses or physicians	Implemented solutions
Mothers and their companions failing to observe the hygiene regulations	‑ Provision of healthcare regulations ‑ Proper orientation of mothers and companions on infection prevention
Mothers failing to observe the rules on leaving the ward	‑ If the mother has to leave the ward for personal reasons, she should take the baby to the neonatal ward ‑ A mother in poor health is allowed to have a companion in the KMC room
Noise pollution (e.g. mothers speaking loudly)	‑ Mothers were informed about the impact of noise pollution on infants ‑ Cell phones had to be on mute and mothers were asked to speak slowly and keep their voices down
Interference with staff members while performing their tasks and duties (e.g. mothers making comments about the nursing procedures while staff were busy with their care)	‑ Mothers were asked to not to interfere with the nursing care
Interruption in care services due to the physicians’ visits to the mothers	‑ Advising the physicians on the issue ‑ Coordinating physicians’ visiting times to the KMC unit
Failure of nurses to respond timeously to mothers’ needs	‑ Reinforcing nurses’ awareness of the importance of responding to mothers’ requests and needs
Mothers’ concerns: ‑ fear of harming the infants during KMC ‑ fatigue due to lengthy care process	‑ Emphasizing the benefits of KMC ‑ Encouragement to provide KMC ‑ Regular monitoring of adherence during the first days of care ‑ Responding to mothers’ questions
Limited presence of fathers (once or twice) in the ward to assist with KMC	‑ Allowing fathers to be present when the ward is less crowded ‑ Providing privacy by means of a room divider (paravan)

### Step 5: evaluating the implementation

Based on the results of applying the progress-monitoring tool, the following are some of the post-implementation observations:

1. A physical space was allocated and the equipment and facilities required for a C-KMC unit were provided.
2. Better sensitization and orientation of mothers were achieved by means of various media (e.g. face-to-face instruction, videos, pamphlets and posters).
3. More mother–infant pairs were able to do KMC because additional space was provided.
4. It was possible to apply KMC for longer periods because of non-separation of mothers and babies.
5. Training was provided for all staff offering care to mothers and infants.
6. Documentation was improved (e.g. a data registration file was created in the neonatal ward).
7. C-KMC guidelines and a training curriculum were drafted with the assistance of the Ministry of Health and Medical Education.

On the progress-monitoring model, the C-KMC program obtained an implementation score of 27.05 out of 30 points (see Table 3). The program lost points on resource allocation policies, transportation of infants in the skin-to-skin position, and discharge and follow-up (and by implication the potential to identify infants for readmission).

**Table 3:** Progress scores for the implementation of the C-KMC program

Stages and phases		Points per stage	Score obtained
Pre-implementation phase			
Stage 1	Create awareness	2	2.00
Stage 2	Commit to implement	2	2.00
Implementation phase			
Stage 3	Prepare to implement	6	5.50
Stage 4	Implement	7	6.50
Institutionalization phase			
Stage 5	Integrate into routine practice	7	6.25
Stage 6	Sustain practice	6	4.80
TOTAL		30	27.05

### Step 6: institutionalizing C-KMC services

For this step, focus group sessions were held and feedback on the evaluation results was given to participants, other interested parties and policy makers in the Ministry of Health and Medical Education. The ideal was to address the weaknesses and challenges in the implementation of C-KMC at all levels through supervision and support with a view to enhancing and institutionalizing KMC services.

## Discussion

This participatory action research project was conducted as part of the launch of a C-KMC unit in a teaching hospital in Iran. It is very important to lay a good foundation when attempting to introduce changes in an organizational environment. Furthermore, enabling interested individuals to participate in the creation of change helps decrease resistance to change and facilitate the conditions required for the change [22]. In this study, the grassroots problems and barriers were identified through the participation of mothers, physicians, nurses and other primary personnel involved in C-KMC and appropriate solutions were sought while designing and executing the C-KMC program. Table 4 gives a summary of key lessons from this participatory action research project.

**Table 4:** Key lessons from this study

Implementation issue	Lessons
Vital elements for successful implementation	• Orientation and education of mothers • Presence of the father • Orientation and training of healthcare personnel aimed at learning and behavior change
Space	The design of the physical space of a KMC ward should • take cultural issues into account • enable the involvement of fathers in C-KMC*
Homely atmosphere in the C-KMC ward	• Mothers need to be kept occupied** • A small library and a TV set can contribute to countering boredom
Ward routines	• Re-visit ward routines to consider mothers’ and babies’ needs and not only those of the physician
Health insurance	• Involve financial managers and insurance administrators in the implementation process

* In our study fathers could only enter the KMC ward if there were no other women in the ward

** The program went well when there were several mothers in the ward or the mother was used to reading or doing handwork (e.g. knitting or embroidering)

In addition to identifying and investigating the needs, barriers and problems involved when offering C-KMC services, the action research in the present study entailed the experimental design and implementation of a program, and reflection on and evaluation of the program. The analysis of the staff interviews revealed numerous barriers to the implementation of C-KMC that prevented the consistent and regular practice of KMC. In order to facilitate optimal C-KMC practice, great emphasis should be placed on the orientation and education of parents, physicians, nurses and other healthcare personnel. In this study it was also necessary to adapt patients’ payment requirements in some cases and address these in the health insurance system [21].

Many studies have emphasized the role of education and training in the creation of appropriate strategies for KMC implementation [23–25]. Continuous in-service education and training for staff were also a key part of this study. However, although passive transfer of information to staff influences the accumulation of knowledge and awareness, it does not necessarily result in improvement of care performance [26]. Attention to educational strategies to enhance deep learning and behavior change is going to be an important part of the further scale-up of C-KMC in Iran.

Although the KMC progress-monitoring tool is more frequently used in measuring progress with KMC implementation across a number of hospitals [27–32], it can also be used for evaluating progress at individual hospitals, as was the case in this study. The implementation score of 27.05 out of 30 obtained in the evaluation following the implementation of C-KMC demonstrated that the hospital was on the path to institutionalizing KMC and integrating KMC into the routine care of small babies [18]. The progress score in the study hospital compares well with the individual scores of centers of excellence in other countries and is better than the highest scores obtained in various other studies. For example, in a Mali study of seven centers, the highest score of 24.57 was achieved by a teaching hospital [33]. The highest scores in two South African implementation studies were 22.94 and 23.79, respectively [27, 28], whereas in a scale-up study in Ghana the highest score was 20.69 [29]. Achieving high implementation scores for teaching hospitals is of great importance because these centers play a major role in information transfer, training and instruction and in demonstrating how KMC should be rendered effectively and efficiently.

The major gaps identified by the evaluation of progress in the study hospital were the acquisition of funding, follow-up practices and the challenge of transporting premature infants in the KMC position. Improving service quality and stability requires resources [34]. Centers planning to offer KMC services should have the necessary budget for human and technical resources, including changes in infrastructure and equipment procurement and maintenance [11, 34]. In the present study, all the costs of equipment, the creation of a physical space, support for mothers and staff training were sourced from the existing hospital budget. When planning to implement C-KMC services at different hospitals and at all levels of care, financial investment may require more resources than individual centers are able to provide.

Appropriate follow-up systems for KMC babies after discharge from hospital and the keeping of proper records of follow-up visits are major challenges globally [19, 31–33, 35]. Regular primary and special follow-up care visits must be scheduled [2, 6]. Iran’s country guidelines provide for a regular follow-up program for premature infants up to the age of four years. The major challenge identified by this research was the nonexistence of a coherent follow-up system. Long trips for parents to attend follow-up, physicians’ preference for conducting follow-up visits in their personal offices and the absence of a follow-up status examination clinic equipped with an integrated recording system in the study hospital are amongst the main reasons for the inadequate provision of appropriate follow-up services. During the feedback discussions, it was agreed that the individual(s) responsible for the KMC room should educate mothers on the importance of follow-up examinations after discharge and should contact mothers at certain times to remind them to come for a follow-up visit.

An infant sometimes needs to be transferred from one ward to another or from one hospital to another to undergo diagnostic examinations and treatment. Transporting infants in the skin-to-skin kangaroo position is the best option and causes less stress [36]. As KMC is still relatively new in the care system for premature infants, there were no facilities for transferring babies to the study hospital in the KMC position. However, a recommendation on operationalizing the transfer of babies in this position was made to hospital officials.

The present study was subject to a number of limitations. The coronavirus pandemic influenced the provision of C-KMC services in the early days of the outbreak in 2020. After the World Health Organization proclaimed a pandemic, Iranian health decision makers – in an attempt to prevent the spread of the disease – ordered the suspension of all elements of family-centered care, including the presence of mothers in NICUs and the C-KMC unit, as well as prohibiting breastfeeding. The research team liaised with the Ministry of Health and Medical Education and the C-KMC unit to find a way forward. Although the Ministry allowed limited KMC and breastfeeding after a four-month assessment of evidence, the study unit was permitted to resume C-KMC after only two months, but with fewer mother–infant dyads admitted to the ward (two instead of four mothers).

A further limitation of the study was the fact that the final evaluation was done by the research team implementing C-KMC. The ideal would have been to have external assessors conduct the progress monitoring. On the other hand, the research team gained valuable insights by conducting the progress monitoring themselves and this learning experience has prepared them to repeat this exercise in other hospitals that are embarking on the implementation of C-KMC.

## Conclusion

It appears that participatory action research was a useful and suitable method for creating change in the healthcare and treatment system through the integration of C-KMC into the continuum of the care of small newborns. The flexibility associated with action research and its approaches allowed for newly-emerging issues to be addressed immediately, assisted with the translation of knowledge into practice in real life, promoted acceptance of KMC as a beneficial form of care for premature infants, and made it possible to observe the improvement in the health outcomes of these vulnerable newborns. Key factors associated with the sustainability of C-KMC unit activities were the collaboration of neonatologists and nurses and mothers` preference for doing C-KMC.

## Data Availability

The datasets used and/or analyzed during the current study are available from the corresponding author on reasonable request.
